# Transcriptional enhancers: functional insights and role in human disease

**DOI:** 10.1016/j.gde.2015.08.009

**Published:** 2015-08

**Authors:** Irene Miguel-Escalada, Lorenzo Pasquali, Jorge Ferrer

**Affiliations:** 1 Department of Medicine, Imperial College London, London W12 0NN, United Kingdom; 2 Genomic Programming of Beta-cells Laboratory, Institut d’Investigacions August Pi i Sunyer (IDIBAPS), 08036 Barcelona, Spain; 3 CIBER de Diabetes y Enfermedades Metabólicas Asociadas (CIBERDEM), 08036 Barcelona, Spain; 4 Division of Endocrinology, Germans Trias i Pujol University Hospital and Research Institute and Josep Carreras Leukaemia Research Institute, 08036 Barcelona, Spain; 5 Josep Carreras Leukaemia Research Institute, 08036 Barcelona, Spain

## Abstract

In recent years, studies of *cis*-regulatory mechanisms have evolved from a predominant focus on promoter regions to the realization that spatial and temporal gene regulation is frequently driven by long-range enhancer clusters that operate within chromosomal compartments. This increased understanding of genome function, together with the emergence of technologies that enable whole-genome sequencing of patients’ DNAs, open the prospect of dissecting the role of *cis*-regulatory defects in human disease. In this review we discuss how recent epigenomic studies have provided insights into the function of transcriptional enhancers. We then present examples that illustrate how integrative genomics can help uncover enhancer sequence variants underlying Mendelian and common polygenic human disease.

**Current Opinion in Genetics & Development** 2015, **33**:71–76

This review comes from a themed issue on **Molecular and genetic bases of disease**

Edited by **Dan E Arking** and **Johanna M Rommens**

For a complete overview see the Issue and the Editorial

Available online 6th October 2015

**http://dx.doi.org/10.1016/j.gde.2015.08.009**

0959-437X/© 2015 The Authors. Published by Elsevier Ltd. This is an open access article under the CC BY license (http://creativecommons.org/licenses/by/4.0/).

## Introduction

Massive sequencing technologies have demonstrated an extraordinary power to uncover disease-causing variants in protein-coding sequences. It is now necessary to ask whether similar technologies can be exploited to discover defects in the ∼1 million transcriptional regulatory sequences that have been unearthed in the past few years. This challenge, however, is hindered by our incomplete understanding of the function of transcriptional regulatory elements. This review will focus on recent advances in understanding the function of transcriptional enhancers, and present examples of how integrative genomics can help identify enhancer defects that underlie Mendelian and polygenic disease.

## Clustering of active enhancers

Enhancers were first defined as DNA sequences that stimulate transcription from a minimal promoter, regardless of orientation or relative distance [1]. Subsequent studies showed that long-range enhancers are pivotal for spatial and temporal regulation of gene transcription in metazoan genomes. Most recently it has become possible to catalogue the entire genomic repertoire of active enhancers in any cellular population by exploiting distinctive enhancer features such as: increased accessibility to enzymes (DNAse-seq, ATAC-seq), or relative nucleosome depletion (FAIRE-seq) [2, 3, 4]; enrichment of specific modified histones (H3K27Ac, H3K4me1) [3, 5]; occupancy by co-regulatory factors (p300, BRD4, Mediator) [6, 7]; and finally, RNA transcription from enhancer-flanking regions [8]. All such features can now be studied with high-throughput sequencing-based assays, which has enabled the generation of enhancer maps in numerous cell lines and primary tissues [8, 9••, 10, 11].

A recurrent theme that emerged from recent enhancer maps is that most lineage-specific gene transcription occurs near clusters of active enhancers. This had been previously recognized in the form of clusters of evolutionary conserved sequences flanking lineage-specific regulatory genes [12], or from functional studies of numerous individual loci. However, enhancer maps now provide an unbiased perspective based on genome-scale experimental data. Regulatory clusters have thus been described as clusters of open regulatory elements (COREs) [2], superenhancers [13], stretch enhancers [14], or enhancer clusters [11]. One study mapped human pancreatic islet enhancers, and found that most islet-enriched genes are associated with three or more clustered enhancers, which tend to be co-occupied by multiple islet-specific TFs [11]. Chromatin conformation capture (3C) assays showed that clustered enhancers form higher order physical structures and establish physical interactions with target genes [11]. These enhancer clusters were consistent with earlier studies showing COREs (open chromatin clusters defined by FAIRE-seq) near islet-specific genes [2], and with the more general observation that expression of genes across multiple tissues correlates with the activity of multiple local enhancers in the same locus [15]. Another study defined ‘stretch’ enhancers as H3K27Ac-rich chromatin regions >3 kb, and found them to be frequently located near cell-specific genes [14]. Another set of studies defined ‘superenhancers’ as extended enhancer regions that show unusually high occupancy by either Mediator, TFs, or H3K27Ac-modified nucleosomes [13, 16••] (for an in-depth commentary on superenhancers see [17^••^]). Superenhancers have been linked to genes that are central for pluripotency or cell type identity as well as to oncogenes, and shown to be particularly sensitive to targeting by co-regulator inhibitors [13, 16••, 18].

Regardless of varying definitions and nomenclatures, recently described enhancer domains are, in essence, sets of adjacent active enhancers. This raises the question of why there is a need for multiple enhancers to create cell-specific transcription. Possible explanations include redundancy (‘shadow’ enhancers) and combinatorial or synergistic specificity, although recent genetic studies provide further explanations. Spitz and colleagues, for example, used a broad range of mouse genetic tools to dissect an enhancer cluster regulating *Fgf8* [19^•^]. This showed that the regulatory output of an enhancer cluster (in this case the cell types in which *Fgf8* is expressed) is not simply a summation of individual enhancer activities, but is instead dependent on a combined function of clustered enhancers, or ‘holo-enhancer units’ [19^•^]. On the other hand, multiple studies indicate that enhancer clusters form higher-order 3D structures [11, 20, 21, 22, 23•], suggesting that ‘holo-structures’ might be crucial for cell-specific transcription.

## Enhancer function in the context of the 3D genome

3C studies have established general principles that underlie 3D genome organization, and promise to enlighten how enhancers interact with their functional targets. Hi-C sequencing has shown that the genome is packaged at multiple organizational levels, including so-called topologically associated domains (TADs) [24^••^]. TADs, which span on average ∼0.8 Mb, are defined by a high number of intra-domain 3C interactions and rare interactions between adjacent domains. A recent study used random insertions of a reporter that acts as a sensor of endogenous enhancer activity, and showed that TADs provide a spatial compartment within which enhancers interact functionally (and not solely physically) with their target promoters [25]. Others have demonstrated coordinated gene regulation within the confines of TADs [26, 27].

Increased resolution mapping using 5C or Hi-C libraries revealed further subdomains within TADs, including ‘loops’ that are bound at their stem by CTCF, as well as cohesin and mediator-bound cell-specific ‘loops’ that link enhancers to promoters [28, 29]. 4C-seq studies, a 3C variant that interrogates all genomic sites interacting with a viewpoint of interest at very high resolution, have shown that clusters of lineage-specific enhancers establish frequent interactions amongst themselves and with target gene promoters [11, 20, 21, 22]. Interestingly, while TAD boundaries are typically invariant across cell types, they contain structures that are often cell-specific and dynamic [28, 30].

Looping into promoters is thought to underlie enhancer function, and this was recently tested by artificial tethering of an enhancer to a promoter, leading to increased transcriptional activity [31]. It is nevertheless also true that each enhancer often shows 3C interaction signals with multiple nearby enhancers and promoters, and each promoter with multiple enhancers and promoters [32, 33]. One theoretical implication of this observation is that if all such interactions are functional, then sequence variation in single enhancers could potentially impact multiple genes. However, while 3C assays most probably do capture regulatory interactions between enhancers and promoters, it is unclear if all 3C interactions are functional. In fact, studies have challenged the significance of 3C interactions, and questioned whether other variables apart from physical proximity affect ligation frequency in 3C experiments, and whether 3C interaction signals represent discrete loops [34]. This warrants a need for crosslink-independent methods for studying 3D structure. Interestingly, a recent study used high-resolution live cell imaging to show widespread Sox2-bound clustered enhancers in ESCs, providing further independent evidence that enhancer clusters form structural units [23^•^]. Diverse approaches are thus becoming available to probe the impact of enhancer mutations on higher order chromatin structures.

Taken together, recent studies provide an initial framework for understanding how long-range enhancers operate in the context of genome organization. Future studies that couple 3D interaction experiments with functional perturbations, including targeted mutations and eQTL studies, should provide further light on mechanistic and functional relationships between enhancers and target genes. This type of knowledge will be vital for understanding how enhancer variants could be deleterious in the context of 3D chromosomal structure, and to identify the genes that are affected by defective enhancers.

## Mendelian regulatory defects

Notable examples of long-range enhancer mutations that cause monogenic disorders include those regulating *SHH* (preaxial polydactyly) [35], *SOX9* (Pierre Robin Syndrome) [36], and *TBX5* (congenital heart disease) [37]. These and other known enhancer mutations were identified after careful functional characterization of enhancers, followed by targeted sequencing, or else by the discovery of large deletions or rearrangements that were subsequently shown to contain enhancers. This approach is relatively inefficient when compared with the success of whole-exome sequencing for detection of protein-coding mutations.

**Figure 1:**
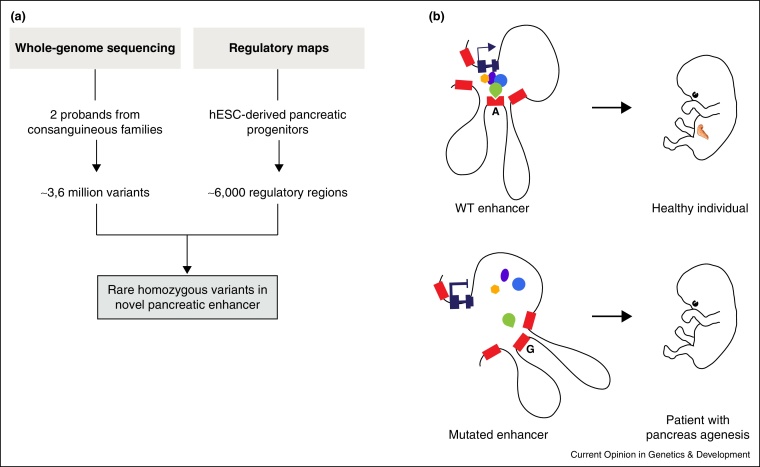
Integrative genomics reveals that isolated pancreas agenesis is caused by enhancer mutations. **(a)** Integration of whole genome sequences from two patients with pancreatic agenesis with maps of active enhancers profiled in human embryonic pancreatic progenitors identified causal recessive mutations. These mutations map to a previously unannotated enhancer 25 kb away from *PTF1A*, a transcription factor that is known to be essential for the embryonic development of the pancreas. **(b)** Schematic representation of the *PTF1A* locus harboring wild-type (A) and mutated (G) enhancer sequences. The newly identified enhancer (indented red box) establishes a physical interaction with the *PTF1A* promoter and is bound by regulatory factors such as FOXA2 (green teardrop). The presence of a single-nucleotide enhancer variant in some patients with pancreatic agenesis (g.23508437A > G) disrupts binding by FOXA2, abolishes enhancer activity and potentially alters the local chromatin structure of the enhancer cluster. A deletion of this enhancer region or other single base mutations that disrupt binding of FOXA2, PDX1 or an unidentified binding protein cause the same phenotype, thus highlighting a crucial role of this enhancer in the active conformation of the *PTF1A* locus.

A recent study exemplifies a systematic approach to discover enhancer mutations (Figure 1). Hattersley and colleagues carried out whole-genome sequencing and homozygosity mapping of SNPs in two unrelated consanguineous probands with isolated pancreas agenesis and no causal protein-coding mutations [38^••^]. Integration of this data with enhancer charts from human embryonic pancreatic progenitors revealed homozygous point mutations in a single unannotated enhancer >25 kb from *PTF1A*, a known pancreatic regulatory gene. Subsequent analysis of 12 unrelated families with isolated pancreas agenesis showed that 10 had rare homozygous mutations in this enhancer, including a large deletion and point mutations that disrupted functional binding sites of pancreatic developmental TFs [38^••^].

The analysis of isolated pancreas agenesis has noteworthy implications. One is that it illustrates how one can progress from a person's inventory of >3 million non-coding variants to the identification of a causal non-coding mutation. It was also an unbiased genome-scale analysis that showed that mutations that disrupt recognizable *cis*-regulatory sequences can be the most common cause of a discrete phenotype (in this case isolated pancreas agenesis). It is also noteworthy that the pancreatic progenitor enhancer that harbored mutations was inactive in a broad panel of tissues, which highlights that any search for non-coding defects needs to focus on disease-relevant epigenomic annotations. Finally, it is interesting that despite that there are multiple pancreatic progenitor enhancers near *PTF1A* [4, 39], all mutations fell in a single enhancer. Analogously, engineered mutations in enhancer clusters show that only some clustered enhancers in *Sox2* are essential in ESCs [40]. This suggests a functional hierarchy within enhancer clusters, perhaps due to a hub-like function of specific enhancers within 3D structures.

The pancreas agenesis studies support future efforts to integrate whole genome sequencing with regulatory annotations to discover Mendelian non-coding defects. Further discoveries of pathogenic enhancer mutations from screens of natural and engineered variants should inform computational algorithms that enable prediction of pathogenic regulatory variants. Despite the limited amount of data, several approaches have already been developed to predict which non-coding variants within regulatory elements are functional. Most have examined whether variants affect nucleotides in TF-binding motifs, are under evolutionary selective pressure, or show polymorphism in humans [41, 42, 43]. Additional factors, such as the position of variant enhancers in the context of regulatory domains, are also probably to affect pathogenicity. The availability of large numbers of regulatory mutations should thus facilitate future understanding of Mendelian and complex non-coding defects.

## Common variation in enhancers and human disease

Most common diseases, including prevalent forms of cancer, Type 2 diabetes, or late-onset Alzheimer's disease, result from environmental factors interacting with genetic susceptibility variants. Genome-wide association studies (GWAS) have identified thousands of loci that affect the susceptibility to common diseases. Many risk loci do not contain causal protein-coding variants, suggesting a role for regulatory variation [44, 45•, 46]. This entails major challenges for translating GWAS findings to molecular insights. Associated haplotype blocks include many variants, which means that it is necessary to identify the specific causal regulatory variants at each associated locus. Even after prioritizing functional variants, there is no straightforward approach to conclusively establish the genes that are affected by the variant, and the relevant biological context. Only when this information is available is it possible to study how inherited changes in gene regulation affect cellular pathways that underlie disease.

Recent studies have made considerable progress to address these challenges. A plethora of studies have now shown that variants associated with common diseases are enriched in enhancers, and this has sometimes led to identification of functional variants [9••, 11, 16••, 44, 45•, 47••, 48]. Studies have further revealed a specific enrichment in enhancer clusters or superenhancers that are active in cell types that match a coherent pathophysiological model of the disease [11, 14, 16••, 49]. Two studies, for example, showed that SNPs associated with Type 2 diabetes and fasting glycemia levels are enriched in pancreatic islet clustered enhancers and stretch enhancers [11, 14]. This showed that islet-specific regulatory variation is relevant to Type 2 diabetes pathophysiology, and enabled functional characterization of discrete risk variants that disrupt TF-binding motifs and impact the activity of islet enhancers [11]. Another example focused on 21 autoimmune disorders, and used dense genotyping of large patient cohorts to greatly reduce the number of candidate causal variants per locus [45^•^]. These SNPs were enriched in non-synonymous protein-coding variants, but also in enhancer variants, with a notorious enrichment in dynamically stimulated and clustered T-cell enhancers [45^•^]. Taken together, recent work has shown that it is possible to identify disease-associated functional variants in cell-specific enhancers, which represents a giant step towards understanding molecular mechanisms of common diseases.

In addition to identifying functional variants, it is challenging to define which genes are affected. A study that analyzed *FTO*, the major obesity susceptibility locus, provides a paradigm for how this problem can be tackled [50^•^]. Risk variants in *FTO* intronic regions were presumed to affect *FTO*, whose mouse KO phenotype causes reduced body size [51, 52]. Unexpectedly, *FTO* regions carrying risk variants form 3C interactions with *IRX3*, located >400 kb away, and confer enhancer activity in cellular domains that coincide with *IRX3* (rather than *FTO*) expression [50^•^]. Furthermore, risk SNPs at *FTO* were associated with brain eQTLs that affected expression of *IRX3*, but not *FTO*. Interestingly *IRX3* KO mice show decreased lean body mass [50^•^]. This landmark study therefore shows that contrary to previous assumptions *IRX3* is a functional target of regulatory variants that affect obesity susceptibility.

In summary, recent studies have started to deploy a broad range of genetic and functional tools that enable untangling the regulatory function of common disease-associated variants. Clearly, the level of evidence that is needed to conclusively implicate a specific non-coding variant in causality remains a challenge. Importantly, most common diseases are not easily modeled in an organism by introducing a single regulatory allele. However, as in classic protein-coding Mendelian diseases, it should be possible to implicate a genetic variant by combining multiple lines of evidence, including human genetics (fine mapping, transethnic studies) and functional studies (allelic expression, reporter and 3C assays, genome editing). Ultimately, the goal is not solely to determine which variants are causal, but to understand the genetic pathways they regulate and to harness this knowledge to develop more efficient therapies.

Until very recent times, the fields of gene regulation and human genetics were largely unlinked. The studies we have reviewed illustrate how recent advances in these two fields are rapidly converging to enlighten new genetic mechanisms underlying Mendelian and polygenic human disease.

## References and recommended reading

Papers of particular interest, published within the period of review, have been highlighted as:

• of special interest
•• of outstanding interest
